# Application of deep learning in the real-time diagnosis of gastric lesion based on magnifying optical enhancement videos

**DOI:** 10.3389/fonc.2022.945904

**Published:** 2022-08-05

**Authors:** Mingjun Ma, Zhen Li, Tao Yu, Guanqun Liu, Rui Ji, Guangchao Li, Zhuang Guo, Limei Wang, Qingqing Qi, Xiaoxiao Yang, Junyan Qu, Xiao Wang, Xiuli Zuo, Hongliang Ren, Yanqing Li

**Affiliations:** ^1^ Department of Gastroenterology, Qilu Hospital of Shandong University, Jinan, China; ^2^ Laboratory of Translational Gastroenterology, Qilu Hospital of Shandong University, Jinan, China; ^3^ Robot Engineering Laboratory for Precise Diagnosis and Therapy of Gastrointestinal Tumor, Qilu Hospital of Shandong University, Jinan, China; ^4^ Department of Gastroenterology, Shengli Oilfield Central Hospital, Dongying, China; ^5^ Department of Pathology, Qilu Hospital, Cheeloo College of Medicine, Shandong University, Jinan, China; ^6^ Department of Electronic Engineering, The Chinese University of Hong Kong, Hong Kong SAR, China; ^7^ Department of Biomedical Engineering, National University of Singapore, Singapore, Singapore

**Keywords:** early gastric cancer, image-enhanced endoscopy, convolutional neural network, deep learning, tumor diagnosis

## Abstract

**Background and aim:**

Magnifying image-enhanced endoscopy was demonstrated to have higher diagnostic accuracy than white-light endoscopy. However, differentiating early gastric cancers (EGCs) from benign lesions is difficult for beginners. We aimed to determine whether the computer-aided model for the diagnosis of gastric lesions can be applied to videos rather than still images.

**Methods:**

A total of 719 magnifying optical enhancement images of EGCs, 1,490 optical enhancement images of the benign gastric lesions, and 1,514 images of background mucosa were retrospectively collected to train and develop a computer-aided diagnostic model. Subsequently, 101 video segments and 671 independent images were used for validation, and error frames were labeled to retrain the model. Finally, a total of 117 unaltered full-length videos were utilized to test the model and compared with those diagnostic results made by independent endoscopists.

**Results:**

Except for atrophy combined with intestinal metaplasia (IM) and low-grade neoplasia, the diagnostic accuracy was 0.90 (85/94). The sensitivity, specificity, PLR, NLR, and overall accuracy of the model to distinguish EGC from non-cancerous lesions were 0.91 (48/53), 0.78 (50/64), 4.14, 0.12, and 0.84 (98/117), respectively. No significant difference was observed in the overall diagnostic accuracy between the computer-aided model and experts. A good level of kappa values was found between the model and experts, which meant that the kappa value was 0.63.

**Conclusions:**

The performance of the computer-aided model for the diagnosis of EGC is comparable to that of experts. Magnifying the optical enhancement model alone may not be able to deal with all lesions in the stomach, especially when near the focus on severe atrophy with IM. These results warrant further validation in prospective studies with more patients. A ClinicalTrials.gov registration was obtained (identifier number: NCT04563416).

**Clinical Trial Registration:**

ClinicalTrials.gov, identifier NCT04563416.

## Introduction

Gastric cancer is the third most common cause of cancer-associated deaths worldwide, particularly in Asia (1). Early detection and treatment can cure the disease, with a 5-year survival rate greater than 90% (2). However, the sensitivity of conventional endoscopy with white-light imaging (C-WLI) in the diagnosis of early gastric cancers (EGCs) is only 40% (3). Magnifying image-enhanced endoscopy (M-IEE) techniques, such as magnifying narrow band imaging (M-NBI), have been developed. Two randomized controlled trials reported that white-light imaging combined with M-NBI can increase the sensitivity to 95% (4, 5). If an abrupt demarcation line (DL) is absent, the diagnosis of a non-cancerous lesion may be made with a 99% negative predictive value (NPV). Therefore, M-IEE may reduce the number of biopsies required to detect EGCs in screening endoscopy (6). The use of white-light imaging to detect suspected lesions and M-IEE techniques to diagnose EGC are recommended for screening endoscopy (7). However, because of the natural background of gastric inflammation, a benign lesion under M-IEE is easily confused with early cancer.

Magnifying optical enhancement (M-OE), an M-IEE technique, was developed by HOYA Co. (Tokyo, Japan). This technology combines digital signal processing and optical filters to clearly display a mucosal microsurface pattern (MSP) and microvessel pattern (MVP) (8). Nevertheless, differentiating EGCs from benign lesions is difficult for beginners with an accuracy of approximately 60% (9). Endoscopists often need to employ considerable effort to improve their diagnostic skills, and expertise with suboptimal interobserver agreement is crucial for the use of M-IEE (10).

Currently, artificial intelligence (AI) using deep machine learning has made a major breakthrough in gastroenterology; it uses the gradient descent method and backpropagation to automatically extract specific image features. The detection accuracy in identifying upper gastrointestinal cancer was 0.955 in C-WLI (11). Polyps can be identified in real time with 96% accuracy in screening colonoscopy (12). A multicenter study showed that C-WLI endoscopy can detect 80% of EGCs (13). AI shows an outstanding application in detection. Meanwhile, several studies verified the M-IEE diagnostic model through still images (14, 15). However, studies using EGC video verification are rare, which further simulated clinical practice. A recent study was able to distinguish tumors from adjacent tissues, using EGC videos (16). The comparison of EGC and various gastric benign lesions is missing. In this study, we included various disease phenotypes that need to be differentiated from EGC to develop an M-OE assistance model and test in unaltered full-length videos to ask whether the assistance model alone can meet the clinical need.

## Methods

This study complied with the Standards for the Reporting of Diagnostic Accuracy Studies initiative and the Declaration of Helsinki (17). It was approved by the Medical Ethics Committee of Qilu Hospital of Shandong University.

### Preparation of image training sets

M-OE images between February 2016 and September 2019 were retrospectively obtained from one tertiary hospital to develop an algorithm for the diagnosis of EGCs. All images were captured by magnifying endoscopy (EG-2990Zi; Pentax, Tokyo, Japan) and OE (EPK-I7000; Pentax, Tokyo, Japan) mode 1. The images of different types of devices came from different patients and were taken from different parts of the lesions. The images with the distal cap attachment were strictly selected. Only images that did not affect the judgment of lesions were retained, which the distal cap attachment accounts for no more than 25% of the image area.

**Figure 1:**
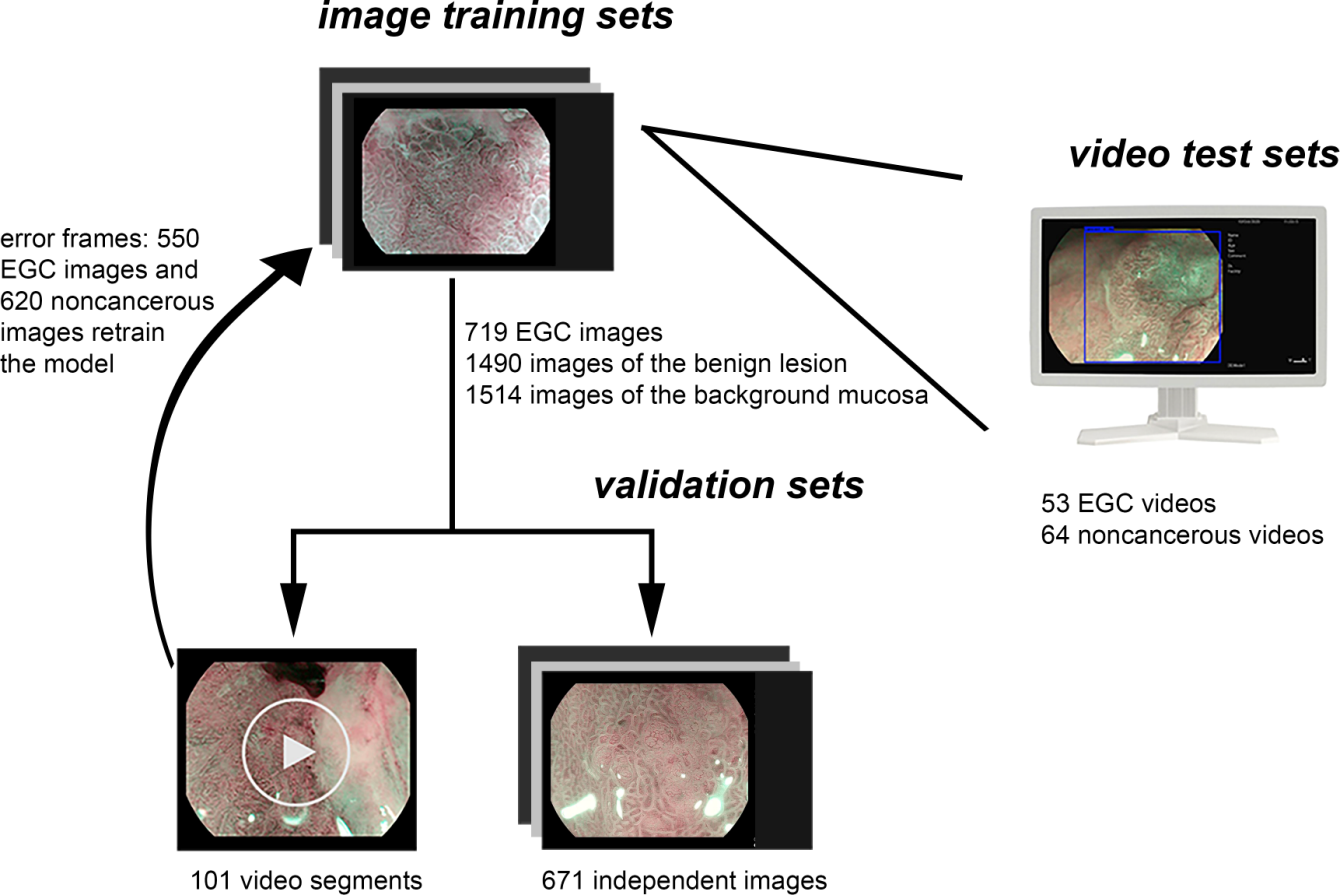
Data flow chart of the computer-aided system. Firstly, the model was developed using the training sets. Secondly, the model was evaluated by independent validation sets including videos and images, and the video frames with error were labeled and retrained the model. Finally, the model was tested using independent test video sets.

Due to the fact that the diagnosis of advanced cancer and active ulcer is usually sufficient with the C-WLI endoscopy, the M-OE gastric images of advanced cancer, lymphoma, the active stage of ulcer, and artificial ulcer after endoscopic submucosal dissection were excluded. Poor-quality images resulting from less insufflation of air, post-biopsy bleeding, halation, blur, defocus, or mucus were also excluded. A single pathologist who was blinded to the endoscopic findings reviewed the histopathology. The pathological diagnostic criteria were based on the revised Vienna classification (18). C4 (mucosal high-grade neoplasia) and C5 (submucosal invasion by neoplasia) were diagnosed as cancer. C1 (negative for neoplasia), C2 (indefinite for neoplasia), and C3 [mucosal low-grade neoplasia (LGIN)] were diagnosed as non-cancerous lesions. Two experienced endoscopists who had performed M-OE for more than 3 years reviewed and manually labeled all images of pathologically proven EGC lesions. LGIN was discarded because a considerable number of LGIN were later confirmed as high-grade or even intramucosal carcinoma (19–21). For cases in which a discrepancy existed between pathological findings and endoscopic diagnosis, the pathologist and endoscopists would reassess and discuss to reach a joint decision. The M-OE images of EGCs should meet the following conditions: the presence of irregular or absence of MVP with a DL or the presence of irregular or absence of MSP with a DL (22). Finally, 952 M-OE EGC images from 126 patients and 3,442 M-OE non-cancerous images from 325 patients were selected. A total of 719 EGC images from 94 patients and 1,490 and 1,514 images of benign lesion and background mucosa, respectively, from 262 patients formed the training set (**Table 1** and **Figure 1**). The rest of the patients and images formed the validation set.

**Table 1:** Image composition of training set.

All cancer	719
EGCs with irregular MSP	571
EGCs with irregular MVP	148
Benign lesion	1,490
Localized intestinal	992
Localized atrophy and scaring	341
Erosion	157
Background mucosa	1,514
Gastric fundus	487
Gastric antrum	1,027

EGCs, early gastric cancer; MSP, microsurface pattern; MVP, microvessel pattern.

### Constructing a convolutional neural network algorithm

You Only Look Once version 4 is a popular and open-access model architecture worldwide (23). It has excellent performance in terms of accuracy and speed.

**Figure 2:**
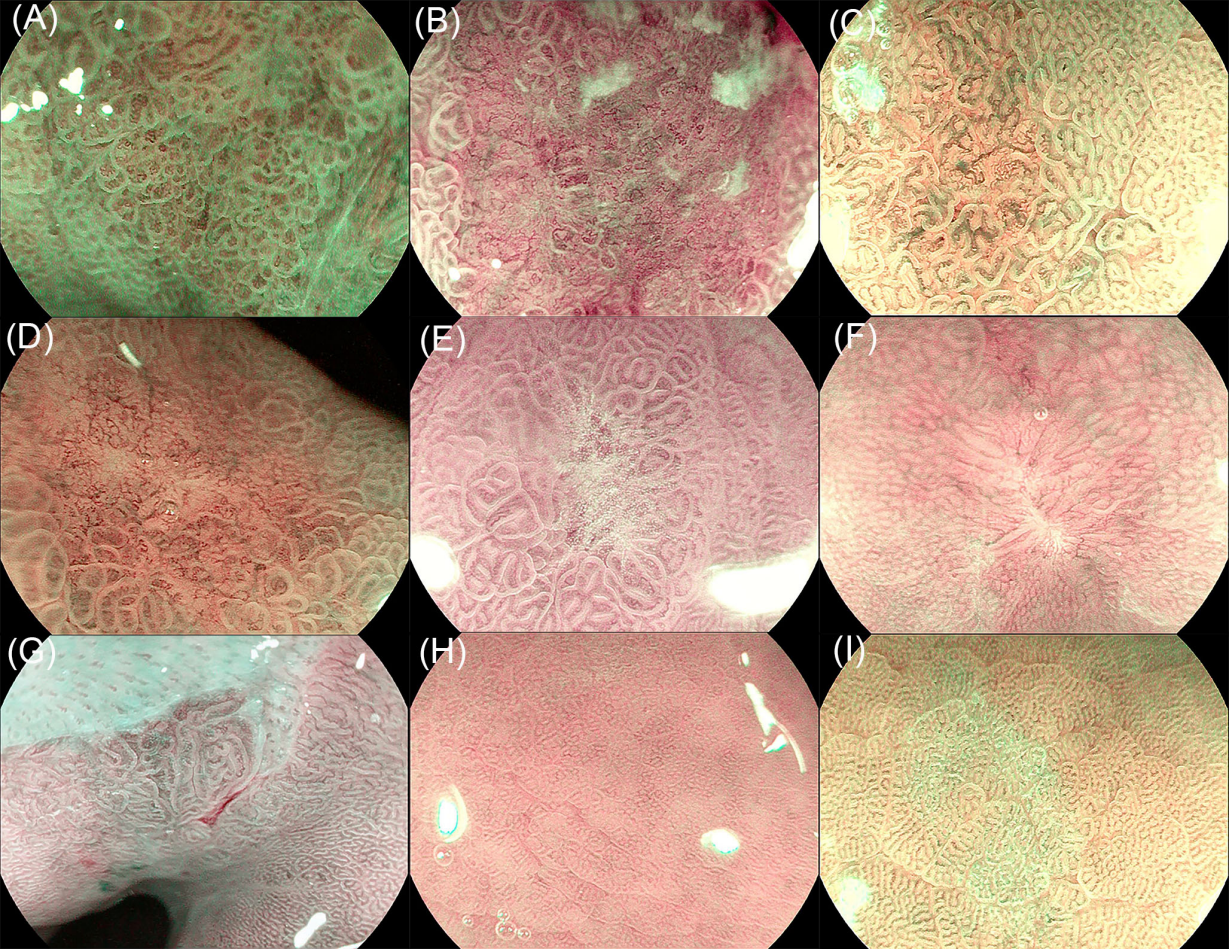
Image classification of the training set. **(A)** Early gastric cancer (EGC) with a mucosal microsurface pattern (MSP), **(B)** early gastric cancer (EGC) with microvessel pattern (MVP), **(C)** localized intestinal metaplasia, **(D)** localized atrophy, **(E)** erosion, **(F)** scaring, **(G)** cardia gland mucosa, **(H)** fundic gland mucosa, and **(I)** pyloric gland mucosa.

Images were divided into three categories: background mucosa, benign lesion, and EGC (**Figure 2**). Background mucosa is a mucosal image of the gastric fundus, cardia, and pyloric gland, which has no suspicious DL. The background images of atrophy and intestinal metaplasia (IM) were also included if there was no suspicious DL. A benign lesion means that the lesion needs to be distinguished from EGCs, such as erosion, localized IM, scarring, and localized atrophy. EGC refers to a cancerous lesion with MVP or MSP. The model automatically analyzes the input images and diagnoses the EGC with a blue rectangular frame and identifies benign lesions and background mucosa with green and red rectangular frames, respectively. For the convenience of statistical analysis, the images of the background mucosa and benign lesions were regarded as non-cancerous images.

An independent set of 671 validation images was prepared from 32 EGC patients and 63 non-cancerous patients, and the model showed 84% accuracy per image analysis. Moreover, 101 video segments recorded in the endoscopic system, 21 EGC video segments and 80 non-cancerous video segments, were used for validation. A total of 550 EGC images and 620 non-cancerous images that were transformed from the error recognition set were manually labeled and retrained.

### Offline unaltered video test sets

A total of 117 full-length M-OE videos recorded using the endoscopy system were included in the test set. All patients had at least one biopsy. If more than one lesion was present in one patient, only the most serious lesion was evaluated.

Our CPU is Intel Core i5-8400, and the GPU is NVIDIA GeForce GTX 1080ti. The processing speed of the model is up to 30 frames/s, which meets the requirements of real-time recognition. (**Supplementary Videos S1**, **S2**) A video set may indicate different diagnostic results for the same lesion, which can be confusing, due to the model output diagnosis to each frame. In order to analyze the diagnostic performance, postprocessing based on videos was performed. The results of the video recognition were concentrated on the time axis, with the cancer tag upward and benign tag downward. The cancer tag would be prompted more frequently for the cancerous lesion. An engineer with no medical background marked out the region of interest (ROI) through automatic calculation by a computer. The ROI started from any cancer recognition tag, and if there was no new cancer recognition tag within 3 s, it was regarded as the end of the ROI. Considering that the longer ROI indicates that the lesion is more likely to be EGC, the total time of the region of interest (TTROI) was recorded.

### Evaluation of endoscopists and outcome measures

A total of 117 full-length videos were used to evaluate the model and experts. Five experts with more than 5 years of experience in magnifying endoscopy were evaluated. In the case of unknown pathology and no interference, the most serious lesion diagnosis was given for each patient.

In the test set, C4 and C5 were considered as cancers based on the gold standard of pathology. The performance of the model was evaluated through the test set. The receiver operating characteristic curve (ROC) was drawn according to the TTROI, and the best cutoff value was obtained. The diagnostic accuracy of each pathological type was analyzed. Sensitivity, specificity, positive likelihood ratio (PLR), negative likelihood ratio (NLR), and overall accuracy were calculated according to the cutoff value.

### Statistical analysis

A Mann–Whitney U test was performed to calculate the statistical difference in the TTROI between cancerous and non-cancerous groups. The Pearson chi-square test was used to determine the statistical difference of no ROI between cancerous and non-cancerous groups. The two-paired McNemar test was performed to compare the accuracy between experts and the model. Kappa analysis was calculated to evaluate the interobserver agreement. Univariate analysis was performed for the pathological type with poor diagnostic accuracy. All statistical tests were two sided, and P<0.05 was considered statistically significant. Analyses were performed using R (version 4.0.2; Vienna, Austria) and SPSS (version 21.0; New York, U.S. IBM Corp).

## Results

A total of 117 full-length videos for 10 h from 64 cases of non-cancerous lesions and 53 cases of EGC were used for the test. The clinical characteristics of the included patients are shown in **Table 2**.

**Table 2:** Clinicopathologic characteristics of gastric mucosal lesions in the test set.

	Early gastric cancer (n=53)	Non-cancerous lesion (n=64)
Age, mean ± SD (range), years	63.3 ± 7.3 (48 – 78)	56.0 ± 10.2 (28 – 78)
Male sex, No. (%)	45 (84.9)	40 (62.5)
Location		
Cardia	12	6
Fundus	12	18
Angle	7	14
Antrum	22	26
Morphology		
0-I	1	
0-IIa	13	
0-IIb	10	
0-IIc	12	
0-IIa+IIc	12	
0-IIb+IIa	3	
0-IIb+IIc	2	
Pathology		
Inflammation		24
Atrophy		10
Intestinal		7
Atrophy + IM		16
Low-grade neoplasia		7
Poor differential EGCs	5	
Differential EGCs	48	

SD, standard deviation; IM, intestinal metaplasia; EGCs, early gastric cancers.

### Model performance

The TTROIs of the cancerous (median, 47 s; interquartile range, 12–90) and non-cancerous groups (median, 0 s; interquartile range, 0–0) were significantly different (*P*<0.0001). No ROIs of the cancerous (9.4%) and non-cancerous groups (78.1%) were significantly different (*P*<0.0001).

**Figure 3:**
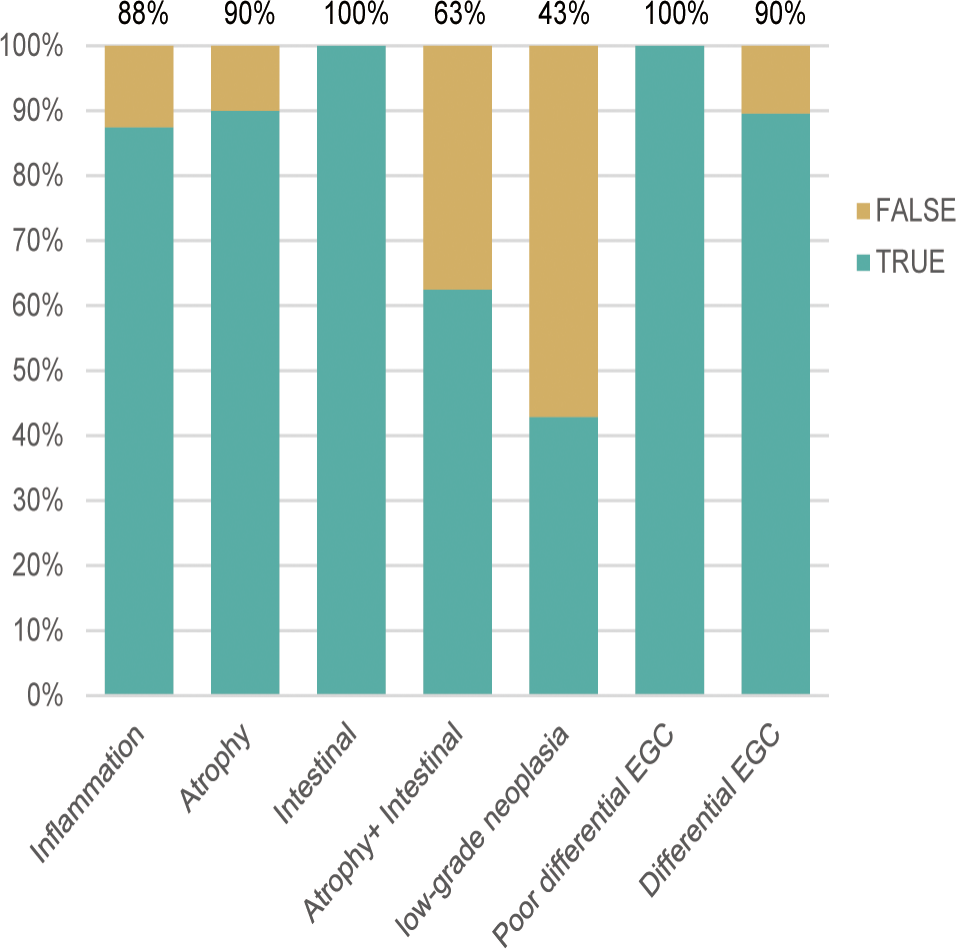
The diagnostic accuracy of different pathological types was approximately 0.90, except for atrophy combined with intestinal metaplasia (0.69) and low-grade neoplasia (0.43). EGC, early gastric cancer.

According to the TTROI, drawing the ROC curve, the area under the curve (AUC) was 0.874 and the best cutoff value was 1 s. Based on the cutoff value, a total of 5 cases of EGC and 14 cases of non-cancerous lesions were misidentified. Except for atrophy combined with IM and LGIN, the diagnostic sensitivity of different pathological types was almost 0.90 (**Figure 3**). The diagnostic sensitivity of atrophy combined with IM and LGIN were 0.63 and 0.43, respectively (**Table 3**). The sensitivity, specificity, PLR, NLR, and overall accuracy of the model to distinguish EGC from non-cancerous lesions were 0.91, 0.78, 4.14, 0.12, and 0.84, respectively.

**Table 3:** Detailed results of the model diagnosis.

	Inflammation(95% CI)	Atrophy(95% CI)	IM(95% CI)	Atrophy+ IM(95% CI)	LGIN(95% CI)	Poor differential EGC(95% CI)	Differential EGC(95% CI)
Sensitivity	0.88 (0.82-0.93)	0.90 (0.85-0.95)	1.00 (1.00-1.00)	0.63 (0.54-0.71)	0.43 (0.34-0.52)	1.00 (1.00-1.00)	0.90 (0.84-0.95)
Specificity	0.83 (0.76-0.90)	0.83 (0.76-0.90)	0.83 (0.76-0.90)	0.87 (0.81-0.93)	0.86 (0.80-0.93)	0.83 (0.76-0.90)	0.80 (0.72-0.87)
PLR	5.09 (1.32-8.85)	5.35 (1.13-9.57)	5.79 (0.77-10.81)	4.86 (1.46-8.25)	3.14 (1.92-4.36)	5.89 (0.67-11.12)	4.42 (1.68-7.15)
NLR	0.15 (0.09-0.22)	0.12 (0.06-0.18)	0.00 (0.00-0.00)	0.43 (0.34-0.52)	0.66 (0.58-0.75)	0.00 (0.00-0.00)	0.13 (0.07-0.19)

CI, confidence interval; IM, intestinal metaplasia; EGC, early gastric cancer; LGIN, low-grade neoplasia; PLR, positive likelihood ratio; NLR, negative likelihood ratio.

### Comparison with endoscopists

No significant difference was observed in the overall accuracy of the diagnosis between the computer-aided model and experts. Nevertheless, the diagnostic accuracy of the model for differential EGCs was significantly different from that of expert C (0.92 *vs*. 0.65, *P* < 0.01). Moreover, although the diagnostic performance of the model for atrophy combined with IM seemed inferior to that of experts, there was no statistical difference between them (0.69 *vs*. 0.94, *P* = 0.13). A good level of kappa values was found between the model and experts, which were 0.62, 0.64, 0.53, 0.61, and 0.71 respectively (**Table 4**).

**Table 4:** Diagnostic accuracy of the model versus experts.

	Inflammation(95% CI)	Atrophy(95% CI)	IM(95% CI)	Atrophy+ IM(95% CI)	LGIN(95% CI)	Poor differential EGC(95% CI)	Differential EGC(95% CI)	Total accuracy (95% CI)	Kappa
Model	0.88(0.69-0.96)	0.9(0.60-0.98)	1.00(0.65-1.00)	0.69(0.44-0.86)	0.43(0.16-0.75)	1.00(0.57-1.00)	0.92(0.80-0.97)	0.84 (0.76-0.89)	
Expert A	0.92(0.74-0.98)	0.9(0.60-0.98)	1.00(0.65-1.00)	0.94(0.72-0.99)*	0.43(0.16-0.75)	1.00(0.57-1.00)	0.9(0.78-0.95)	0.88 (0.81-0.93)	0.62
Expert B	0.92(0.74-0.98)	0.7(0.40-0.89)	0.86(0.49-0.97)	0.63(0.39-0.82)	0.43(0.16-0.75)	1.00(0.57-1.00)	0.92(0.80-0.97)	0.83 (0.75-0.89)	0.64
Expert C	1(0.86-1.00)	0.9(0.60-0.98)	1.00(0.65-1.00)	0.94(0.72-0.99)*	0.43(0.16-0.75)	1.00(0.57-1.00)	0.65(0.50-0.77)^†^	0.80 (0.72-0.87)	0.53
Expert D	0.83(0.64-0.93)	1(0.72-1.00)	1.00(0.65-1.00)	0.94(0.72-0.99)*	0.57(0.25-0.84)	1.00(0.57-1.00)	0.79(0.66-0.88)	0.85 (0.77-0.90)	0.61
Expert E	0.92(0.74-0.98)	0.9(0.60-0.98)	1.00(0.65-1.00)	0.94(0.72-0.99)*	0.43(0.16-0.75)	1.00(0.57-1.00)	0.92(0.80-0.97)	0.90 (0.83-0.94)	0.71

* P = 0.13, ^†^ P < 0.01; CI, confidence interval; IM, intestinal metaplasia; EGC, early gastric cancer; LGIN, low-grade neoplasia.

### Influencing factors for the recognition of atrophy combined with intestinal metaplasia and low-grade neoplasia

We conducted a univariate analysis on factors that may lead to the false diagnosis of atrophy combined with IM (**Table 5**). The proportion of moderate, severe, and diffuse lesions increased in the error group, suggesting that severe diffuse atrophy combined with IM may be difficult to distinguish from EGC by using the computer-aided model.

**Table 5:** Univariate analysis of diagnostic errors of atrophy combined with intestinal metaplasia.

	Correct (n=10)	Wrong (n=6)	P
**Sex**			0.869
Male	8 (80)	5 (83.3)	
Female	2 (20)	1 (16.7)	
**Location**			0.551
Upper	2 (20)	2 (33.3)	
Lower	8(80)	4 (66.7)	
**Severity**			0.037*
Moderate and severe	5(50)	6 (100)	
Mild	5 (50)	0 (0)	
**Range**			0.152
Diffuse	3 (30)	4 (66.7)	
Local	7 (70)	2 (33.3)	

*P < 0.05.

There were seven LGINs in the unaltered full-length video data set, including four ESD-certified LGINs showing high-grade neoplasia in biopsy. Five of the seven LGINs were deemed as having obvious DL by experts, which was an important endoscopic characteristic for diagnosing EGC. Therefore, even the endoscopic experts showed poor diagnostic accuracy for LGIN lesions (0.43–0.57).

## Discussion

We developed an M-OE model covering multiple gastric lesions, using unaltered full-length M-OE videos to evaluate whether the M-OE model can meet the needs of clinical diagnosis. High diagnostic sensitivity was obtained for pathological subtypes, except atrophy combined with IM and LGIN. The overall accuracy is comparable to experts. Currently, three studies on the AI of magnifying EGC are available, all of which were verified by images (14, 15). Considering the variety of benign lesions under magnifying endoscopy, Hiroya et al. (24) included various types of benign lesions in the training set. However, their non-cancerous and cancerous images originated from the same patient. No biopsy pathology was performed; hence, they excluded any suspicious benign lesions to prevent the mixing of cancerous images. A recent video verification of EGC has shown similar results with us (16). However, they used the images of adjacent tissues as the control group and the adjacent tissue of the EGC is often the background mucosa, which could overestimate the performance of the model. In addition, the video clips they used may not represent clinical practice. It is difficult to adapt to multiple gastric lesions only by distinguishing between EGC and adjacent tissues. The distinction between cancer and benign lesions needs further verification. In order to achieve real-time clinical assistance, our study overcomes the shortcomings of the above studies and still achieves a high diagnostic ability through video verification in the case of multiple benign lesions.

We analyzed the statistical distribution of the TTROI and no ROI between cancerous and non-cancerous groups. In the cancerous group, only five cases had no ROI and the median TTROI was 47 s, whereas in the non-cancerous group, 50 cases had no ROI and the median TTROI was 0 s. The TTROI and no ROI were significantly different (P<0.0001). Our model can well distinguish cancerous from non-cancerous groups, which achieve 0.91 sensitivity and minimize the missed diagnosis of EGC. The main reason for cancer recognition errors is differentiated carcinoma with a color similar to that of the background mucosa. We improve the robustness of the model by giving the model different images of benign gastric lesions. For different pathological types, most pathological types are close to 0.90 sensitivity and 0.80 specificity regardless of being benign or malignant.

The diagnostic accuracy of atrophy combined with IM and LGIN needs to be further improved, which causes low specificity. LGIN was also poorly recognized by experts. Since one-quarter of LGIN is diagnosed as EGC after the operation (19–21), a diagnostic resection of LGIN with DL is recommended in our center, which is consistent with the European guideline (18). Meanwhile, there is heterogeneity in the diagnosis of LGIN by pathologists. Therefore, we did not regard LGIN as a kind of lesion with deep learning features alone, to prevent dirty data from affecting the diagnostic efficiency of the model. We speculate that by constantly giving clear malignant and benign data, the final diagnostic efficiency will be generalized to the LGIN pathological entity. At present, the diagnostic accuracy of LGIN by the model is not inferior to that of experts.

Through the analysis of possible reasons for poor recognition of atrophy combined with IM, we found that the model is difficult to accurately diagnose severe and diffuse gastritis. Mistakes usually occur when the lesion is observed at near focus so that the DL of lesions is beyond the visual field, which indirectly indicates that the DL of lesions is an important feature of differentiating benign from malignant. In the practice of endoscopy, it is impossible to ensure that the DL of lesions is always in the endoscopic field, especially for magnifying endoscopy. Combining with the C-WLI detection model may solve this problem (25). Although it could produce false-positive prompts, our model can provide not only a diagnosis of the lesion but also the frame lesion location. In the actual test, endoscopists can make a secondary judgment on the suspicious lesions by the presence or absence of DL and actively discard some false-positive interferences. Therefore, we speculate higher specificity and PPV in practical applications. In any case, AI cannot replace endoscopists but only provide aid in clinical practice.

However, the NLR of our model is 0.12, which indicates that the model could rule out cancer robustly. Another strength is that the positive threshold is only 1 s. If the positive threshold is too high, overlong false-positive prompts will cause trouble for endoscopists in clinical practice. At the same time, the model has a good warning effect on suspected lesions, especially LGIN and severe atrophy combined with IM. The malignant risk of these two kinds of lesions is increased, so timely reminding endoscopists can evaluate these high-risk precancerous lesions more pertinently. Our model can adapt to the unaltered full-length video, which is undoubtedly progressive. Compared with the video clips, the model will encounter more situations that are biased toward clinical practice and needs a low negative likelihood ratio to adapt to long non-cancerous video segments. Of course, our model still needs to be verified online to evaluate the impact of the model on the observation of endoscopists.

This study had several limitations. First, it was conducted in a single center, and the test set is still relatively small. Second, the M-OE model only recognized MSP, MVP, and DL features, excluding C-WLI features. The C-WLI characteristics of the full-length video test will inevitably affect the diagnosis of endoscopists. It is necessary that the model could be applied to other endoscopy systems, especially NBI, which is the most commonly used image enhancement system. We will develop a united model including C-WLI and M-NBI in the future research. Third, the recognition ability of LGIN needs to be further improved. Last, the active ulcer was excluded in this study since the serious inflammatory background and the reactive hyperplasia of the mucosa surrounding the active ulcer would interfere with the model’s diagnosis. Since the combination of C-WLI and M-OE is required to determine whether lesions are benign or malignant, the application of the model is limited.

## Conclusion

We developed a new computer-aided system for the diagnosis of multiple gastric lesions in M-OE endoscopy, which is comparable to that of experts. The M-OE model alone may not be able to deal with all lesions in the stomach. Further development of our computer-aided system will combine the WLI model with the M-IEE models for automatic detection of lesions and accurate real-time diagnosis.

## Data availability statement

The raw data supporting the conclusions of this article will be made available by the authors, without undue reservation.

## Ethics statement

The studies involving human participants were reviewed and approved by Medical Ethics Committee of Qilu Hospital of Shandong University. The patients/participants provided their written informed consent to participate in this study.

## Author contributions

Guarantor of the article: YL. Specific author contributions: MM, ZL, and TY should be considered joint first author. Conception and design: MM, ZL, YL, and TY. Analysis and interpretation of the data: MM, ZL, GQL, RJ, GCL, ZG, LW, QQ, and XW. Drafting of the article: MM. Critical revision for important intellectual content: XZ, HR, JQ, and XY. All authors contributed to the article and approved the submitted version.

## Funding

This study was funded by the National Natural Science Foundation of China (81873550 and 81670489), Key Research and Development Program of Shandong Province (2018CXGC1209). This study is also funded by the Taishan Scholars Program of Shandong Province, Shandong Provincial Key Research and Development Program (Major Scientific and Technological Innovation Project) (2021CXGC010506), and Clinical Research Center of Shandong University (No. 2020SDUCRCC022).

## Acknowledgments

The authors offer sincere gratitude to Xue-jun Shao, Yong-hang Lai, and Jian Feng for the technical support of the artificial intelligence algorithm and Suzette Pearl and Editage (www.editage.cn) for English polishing.

## Conflict of interest

The authors declare that the research was conducted in the absence of any commercial or financial relationships that could be construed as a potential conflict of interest.

## Publisher’s note

All claims expressed in this article are solely those of the authors and do not necessarily represent those of their affiliated organizations, or those of the publisher, the editors and the reviewers. Any product that may be evaluated in this article, or claim that may be made by its manufacturer, is not guaranteed or endorsed by the publisher.

## Abbreviations

C-WLI, white-light imaging; EGC, early gastric cancer; M-IEE, magnifying image-enhanced endoscopy; M-NBI, magnifying narrow band imaging; M-OE, magnifying optical enhancement; DL, demarcation line; IM, intestinal metaplasia; MSP, microsurface pattern; MVP, microvessel pattern; ROI, region of interest; TTROI, the total time of the region of interest; LGIN, mucosal low-grade neoplasia; CNN, convolutional neural network; PLR, positive likelihood ratio; NLR, negative likelihood ratio; ROC, receiver operating characteristic; AUC, area under the receiver operating characteristic curve.
